# Onset Pattern and Long-Term Prognosis in Schizophrenia: 10-Year Longitudinal Follow-Up Study

**DOI:** 10.1371/journal.pone.0067273

**Published:** 2013-06-26

**Authors:** Nobuhisa Kanahara, Taisuke Yoshida, Yasunori Oda, Hiroshi Yamanaka, Toshihiro Moriyama, Hideaki Hayashi, Takayuki Shibuya, Yasunori Nagaushi, Takashi Sawa, Yoshimoto Sekine, Eiji Shimizu, Makoto Asano, Masaomi Iyo

**Affiliations:** 1 Department of Psychiatry, Graduate School of Medicine, Chiba University, Chiba City, Chiba, Japan; 2 Department of Psychiatry, Chiba Psychiatric Medical Center, Chiba City, Chiba, Japan; 3 Division of Medical Treatment and Rehabilitation, Center for Forensic Mental Health, Chiba University, Chiba City, Chiba, Japan; 4 Department of Cognitive Behavioral Physiology, Graduate School of Medicine, Chiba University, Chiba City, Chiba, Japan; Rikagaku Kenkyūsho Brain Science Institute, Japan

## Abstract

**Background:**

Although the duration of untreated psychosis (DUP) plays an important role in the short-term prognosis of patients with schizophrenia, their long-term prognosis generally is not determined by DUP alone. It is important to explore how other clinical factors in the early stage are related to DUP and consequent disease courses.

**Methods:**

A total of 664 patients with untreated psychosis were surveyed for this study. At the first examination, we divided them into the severe positive symptoms cases (SC) or the less severe cases (NonSC) and compared the prognosis among the two groups after a 10-year follow-up. In all, 113 patients in the SC group and 43 patients in the NonSC group were follow-up completers.

**Results:**

Whereas DUP was not different between the two groups, patients with nonacute onset in both groups had significantly longer DUP than those in patients with acute onset. For all clinical measures, there was no difference in prognosis between the two groups or among the four groups classified by mode of onset (MoO) and initial severity of positive symptoms. However, the degree of improvement of global assessment of functioning (GAF) was significantly smaller in the NonSC-nonacute group than in the SC-acute and SC-nonacute groups.

**Conclusions:**

These results suggest that neither DUP nor MoO alone necessarily affects the initial severity of positive symptoms. Moreover, it is possible that patients with low impetus of positive symptoms onset within long DUP experience profound pathologic processes. Therefore, the current study results indicated that long DUP and nonacute onset were related to poor long-term prognosis, regardless of initial positive symptoms.

## Introduction

Schizophrenia is a highly heterogeneous disorder, and its long-term course and/or prognosis also vary significantly from patient to patient. Several studies reported that, although symptom remission could be obtained for 27% of patients within 4 weeks and 45% within 5 years following treatment initiation [1], [2], 20–30% of patients reached a treatment-resistant status on the other side [3]. Even if patients reach remission once, a high relapse rate is inherent in this disorder. Actually, 35% of patients with schizophrenia experienced relapse within 2 years [4] and 74% within 5 years [5] after the onset. These findings indicate that predicting the *true* prognosis of an individual patient requires long-term observation, which exceeds at least the critical period [6].

The prediction of prognosis during the early stage is, nonetheless, importance form the viewpoint of developing the optimal treatment strategy throughout the overall disease course. The authors of previous studies have presented a variety of potential predicting factors, such as duration of untreated psychosis (DUP), mode of onset (MoO) and premorbid functioning [7], [8]. DUP has been reported to have a significant negative impact on various symptoms, remission rate and social functioning in the prognosis of several years after treatment intervention [9], [10]. For long-term outcome, some longitudinal studies have shown that the effect of DUP is not as great as the previously believed [11]. As regard MoO, insidious onset has been suggested to be related with unfavorable outcome [12], [13]. Accumulating evidence of these two factors (DUP and MoO) indicates that their numeric values were not only clinical usefulness, but also that onset pattern could have great effects on the etiology occurring prior to treatment intervention and further consequent disease course. If these two factors already determined severity of symptoms at treatment intervention, the combined analyses based on these three factors (DUP, MoO and initial symptom severity) might be able to anticipate reliably long-term prognosis.

Here, the present study evaluated untreated psychotic patients, who were mostly subjects with schizophrenia, with focus particular on DUP and MoO. Concurrently, we divided them into two groups based on initial severity of positive symptoms and further followed them during 10 years. The reason of selecting positive symptoms as initial severity is that positive symptoms was the most reliable and valid symptom scale among various ones, in particular for first-episode psychosis. The present study aims at clarifying the relationships among predicting factors (DUP and MoO) and initial positive symptoms and further their remote effects on prognosis.

## Methods

### Informed Consents

This study was approved by the ethics committees of Chiba Psychiatric Medical Center (CPMC) and Chiba University. Oral and written informed consents for study inclusion were obtained from all participants and from their family members, if possible, at the prognosis interview. The consents obtained from participants were for 1) conducting an interview with the participant, and 2) using the participant’s clinical data that had been saved at CPMC, to facilitate the appropriate research. When a potential participant denied permission for study participation (even if the denial was judged to be due to his/her illness condition), we neither conducted the interview with the patient’s family nor accessed the patient’s medical records. On the other hand, when a potential participant could not understand our explanations or could not adequately judge whether to participate in the study due to his/her illness condition, following careful discussion by researchers and the patient’s physician, we approached his/her family member (parents or spouse) to discuss the study participation. If the family member agreed to enlist the patient in the study, the interview was conducted with both the patients and the family. As a third case, when the participant agreed to participation in the study, we conducted the interview with the patient and, if possible, with his/her family members. Patients were assured both in writing and verbally that refusal to participate in the study would not have any effect on their subsequent treatments.

### Subjects

For this study, we recruited patients who had received no treatment with antipsychotics or who had received antipsychotics within one week and had not reached sufficient improvement of the relevant psychotic symptoms, which we defined as absence of treatment history, from among patients who had visited CPMC from April 1, 1996 to March 31, 2001. The hospital is an emergency psychiatric center serving all of Chiba prefecture, which has 6 million residents in its catchment area. CPMC is the pioneer hospital of the super-emergency system in Japan, which commits emergency psychosis cases, collaborating with public health departments, rescue teams and police offices. The hospital provides extensive pharmacotherapy and psych-education to patients with acute-stage psychosis and their families. Based on an agreement with other psychiatric hospitals within the prefecture, any patient who lives fairly far from CPMC is treated in the hospital during the acute stage of his/her illness and then transferred into his/her local hospital after some improvement of the disease. CPMC thus manages severe psychotic cases in the most proactive manner amongst psychiatric hospitals in Chiba prefecture.

If patients were diagnosed as suffering from alcohol-related or illegal drug-related psychosis, organic brain or symptomatic psychosis, or psychosis due to any dementia at the first examination or at any time during their follow-up, they were excluded from this study. All participants were diagnosed according to the Structured Clinical Interview for DSM-IV (SCID) [14], by researchers (N.K., T.Y) and by their own physician only at the 10-year follow-up point. Thus, delusional disorder (F24), schizoaffective disorder (F25) and the like, except for schizophrenia (F20) were included in this study.

### Study Design

In the present study we divided participants into two groups. Patients who were judged to be in need of involuntary admission due to profound psychotic symptoms were classified as belonging to the severe case at admission group (SC-group), while patients who received initial treatment intervention in the outpatient clinic were classified as belonging to the non-severe case at admission group (NonSC-group). Voluntary admission based on patient request was impossible, and all admitted cases were involuntary admissions, including medical protection admissions based on requests from patients’ families, or involuntary admissions based on orders from the government.

MoO was assessed for the individual patients at the treatment intervention and thus in the present study we have conducted follow-up for four subgroups based on positive symptoms severity (SC-group, NonSC-group) and MoO (acute onset, nonacute onset). After patients received 10 years of treatment, we evaluated each clinical symptom as long-term prognosis as well as the SCID interview as final diagnosis.

### Assessments

Data at first examination as well as information about improvements following treatment intervention were evaluated through interviews with patients and their families. We analyzed the patient data from the original data base system of CPMC, which was established when the center opened, and thus DUP, MoO and global assessment of functioning (GAF) could be extrapolated by using this system.

DUP is defined as the period between the onset of any psychotic symptom and treatment intervention for the symptom, which led to consequent continuous treatment. As regards MoO, when the patient’s state, which has maintained his/her premorbid function including interpersonal relationships before the onset, deteriorates within about one month, such an onset pattern was judged to be acute onset [13], [15], while other onset cases were judged to be nonacute onset. To ensure there was a clear difference in positive symptoms at the first admission between the groups, we assessed retrospectively the degree of positive symptoms at that time. Positive symptoms (disorganization, suspiciousness, delusion, unusual thought content) and psychomotor excitement at first examination were evaluated if patient symptoms rated a score of 5 or higher on the corresponding item in the Brief Psychotic Rating Scale (BPRS) [16] based on patient medical and nursing records.

To assess patient prognosis, we conducted direct interviews with patients, and when possible, their family members. To measure prognosis, we evaluated BPRS, positive symptoms [17] and negative symptoms [18] from BPRS, GAF and remission level [19]. Those who died from any cause during the follow-up were excluded from the present analysis.

### Statistics Analysis

The statistical procedure was conducted with IBM SPSS Statistic ver. 19 (SPSS Inc., Chicago, IL, USA). Since DUP was extremely positively skewed, the values were transformed into natural logarithms. A chi-square test was applied for categorical variables. For continuous variables in background data, we applied ANOVA when there was equal distribution or the Kruskal-Wallis test when there was not equal distribution. For the analysis of prognosis measurements between groups we performed ANCOVA, with potential factors having effects on prognosis, gender, age of onset, MoO and DUP as covariates.

## Results

**Figure 1:**
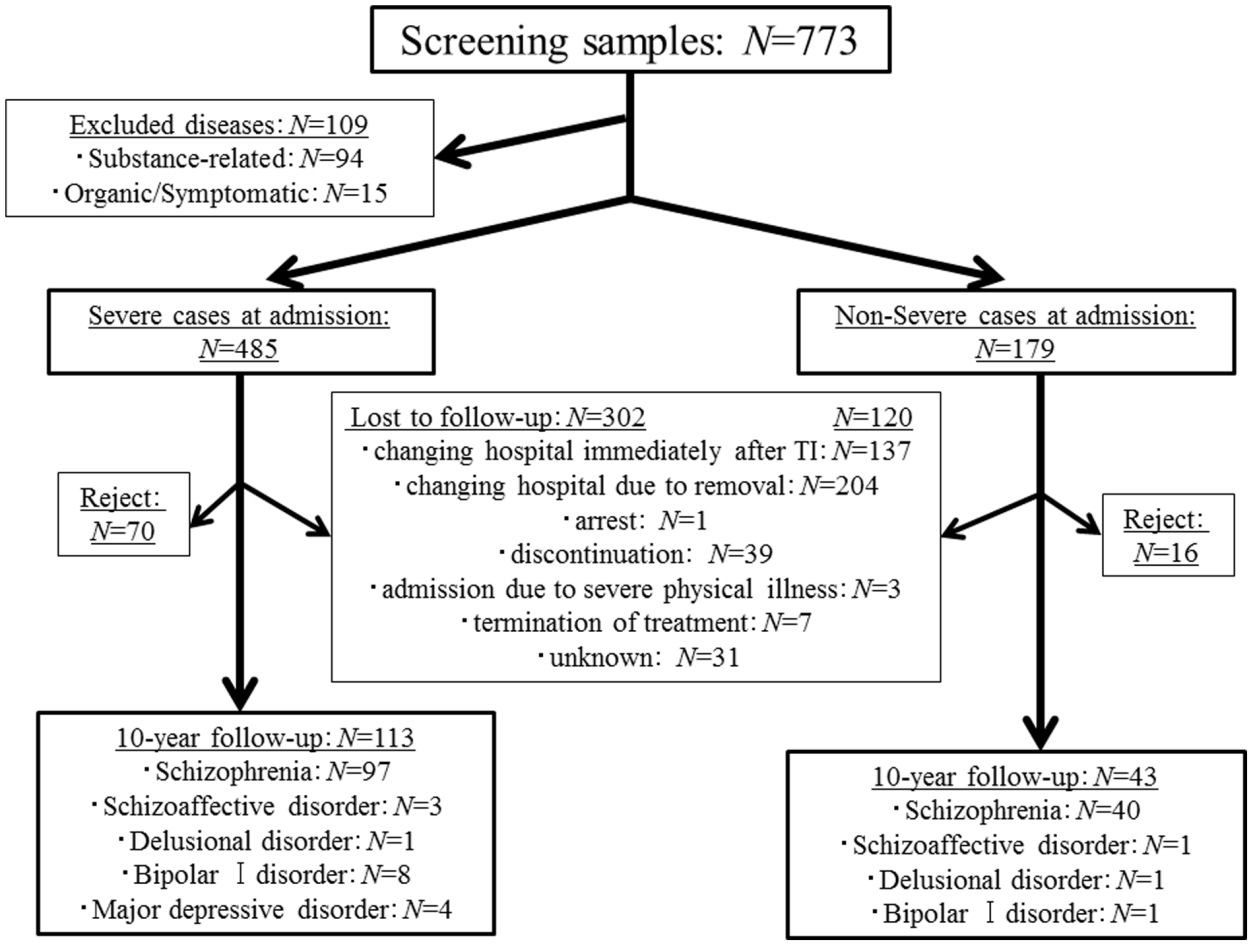
Flow chart of the present study.

We recruited 773 patients with no treatment history. Of these, 109 patients were excluded according to the exclusion criteria. Among the remaining 664 cases, 485 (73.0%) were classified as belonging to the SC-group and 179 (27.0%) were classified as belonging to the NonSC-group (**Fig. 1**). A total of 401 patients had never been medicated, including 282 patients in the SC-group (58.1%) and 119 patients in the NonSC-group (66.5%). Two hundred forty two of the 664 cases (36.4%) received a prognosis interview at the 10-year follow-up. The reasons for dropping out of the study are shown in **Fig. 1**. There were 86 cases who rejected study participation. Therefore, 113 cases in the SC-group and 43 cases in the NonSC-group participated in the final analysis (**Fig. 1**).

### Initial Measurements

#### 1. Comparison of characteristics between the SC and NonSC groups

Gender, age at onset, MoO and DUP did not significantly differ between the SC- and NonSC- groups (**Table 1**). However, GAF in the SC-group was significantly lower than that in the NonSC-group. Furthermore, the SC-group exhibited higher rates of patients with emergency requests, sedative procedures with drug injection, 5 or greater points in positive symptoms and excitement than those of the NonSC-group.

**Table 1:** Clinical characteristics at admission.

Clinical variables	Severe cases at admission (SC)	Non-severe cases at admission (NonSC)	Statistic values
sex [male/female]	113 [61/52]	43 [16/27]	N.P.^#^
age at onset	34.2 y (11.9)	33.3 y (12.3)	N.P.^□^
emergency request (%)[police/emergency]	50% [33/24]	7% [1/2]	*P*<0.001^#^
positive symptoms scores (≥5)^a^	95%	50%	*P*<0.001^#^
excitement score (≥5)^b^	53%	5%	*P*<0.001^#^
sedation with injectable drug^c^	65 [59%]	4 [10%]	*P*<0.001^#^
mode of onset [acute/nonacute]	29/84	12/31	N.P.^ #^
GAF	16.6 (8.8)	30.7 (7.8)	*P*<0.001 ^□^
DUP mean	22.1 m (43.3)	24.6 m (50.6)	N.P. ^□^
DUP median	4.0 m	6.0 m	

**a**: Rate of the number of patients with 5 or greater points in at least one positive symptoms item within disorganization, suspiciousness, delusion and unusual thought content, among each group.

**b**: Rate of the number of patients with 5 or greater points in excitement item among each group.

**c**: Number of patients who required sedation by injected drugs, including haloperidol and/or fulnitrazepam.

#: Chi-square test, ^□^: Student t-test, *abbreviations*: y = years; m = months; number in parentheses indicates standard deviation.

#### 2. Comparison between follow-up and no follow-up cases

The rate of acute MoO in dropping-out cases (*N* = 302) was higher than that in the follow-up cases (*N* = 113) (*P*<.05) in the SC-group. The other factors did not differ among the two groups. In the NonSC-group, gender rate, age at onset, MoO, GAF and DUP were similar among follow-up (*N* = 43) and drop-out cases (*N* = 120).

### Measurements at 10-year Follow-up

#### 1. Comparison in prognostic variables between the SC and NonSC groups

There were no statistical differences in any variables between the SC- and NonSC-groups, but all of these parameters indicated favoring trends for the SC-group (data not shown). When these analyses were conducted for only patients with schizophrenia, these results did not differ.

There were 12 deceased cases overall during the follow-up period, and 9 of these cases were judged to be due to suicide. All of these cases were included in the SC-group.

#### 2. Comparison in prognostic variables based on classification of positive symptoms and mode of onset

Further analysis was conducted by classification of MoO for both the SC-group and NonSC-group (**Table 2**). Although age did not differ among the groups, DUPs of the SC-nonacute-group and the NonSC-nonacute-group were significantly longer than those of the SC-acute-group and the NonSC-acute-group. Baseline GAF of the SC-acute-group and the SC-nonacute-group was lower in comparison to that of the NonSC-acute-group and the NonSC-nonacute-group.

**Table 2:** Comparisons in baseline and 10-year prognosis between acute onset and nonacute onset in each categorical group.

Clinical variables	Severe cases at admission (SC)		Non-severe cases at admission (NonSC)		Statistic values
	Acute onset	Nonacute onset	Acute onset	Nonacute onset	
	(N = 29)	(N = 84)	(N = 12)	(N = 31)	
*Baseline*					
age at onset	33.1y (10.8)	34.6 y (12.3)	35.9 y (11.4)	32.3 y (12.6)	N.P. ^□^
GAF	16.2 (8.2)	16.7 (9.0)	30.6 (6.1)	30.7 (8.5)	*P*<0.000^$^
					SC-Ac/SC-Nonac<NonSC-Ac/NonSC-Nonac
DUP mean	0.39 m (0.41)	29.6 m (48.1)	3.9 m (10.2)	32.6 m (57.5)	*P*<0.000^$^
					SC-Ac/NonSC-Ac<SC-Nonac/NonSC-Nonac
DUP median	0.25 m	8 m	0.5 m	15 m	
*10-year follow-up*					
duration of follow-up	553.5 d (164.7)	563.9 d (108.6)	609.6 d (79.3)	572.8 d (125.9)	N.P. ^□^
duration of total admission	106.3 d (102.8)	129.5 d (89.3)	9.2 d (19.5)	95.6 d (183.1)	*P*<0.05^$^
					NonSC-Ac<NonSC-Nonac<SC-Ac/SC-Nonac
BPRS total	9.9 (8.4)	14.2 (11.3)	12.0 (11.2)	16.3 (11.5)	N.P. ^□^
BPRS positive	3.2 (3.8)	4.2 (4.3)	3.3 (4.9)	5.2 (4.5)	N.P. ^□^
BPRS negative	1.9 (2.3)	4.0 (4.2)	3.4 (4.4)	4.1 (3.4)	N.P.^ $^
death cases [suicide]	5 [3]	7 [6]	0 [0]	0 [0]	
GAF	58.4 (19.1)	51.9 (17.6)	58.2 (21.8)	47.6 (15.0)	N.P. ^□^
Remission rate	51.7%	35.7%	41.7%	32.3%	N.P.^#^

#: Chi-square test, ^□^: ANCOVA, $: ANOVA *abbreviations*: y = years; m = months; d = days; number in parentheses indicates standard deviation.

With respect to prognostic variables, admission duration of the SC-acute-group and the SC-nonacute-group was significantly longer than that of the NonSC-acute-group and the NonSC-nonacute-group, and the admission duration in the NonSC-nonacute-group was longer than that in the NonSC-acute-group. However, for BPRS total, positive symptoms, negative symptoms, GAF and remission rate, there was no significant difference among the four groups (**Table 2**).

**Figure 2:**
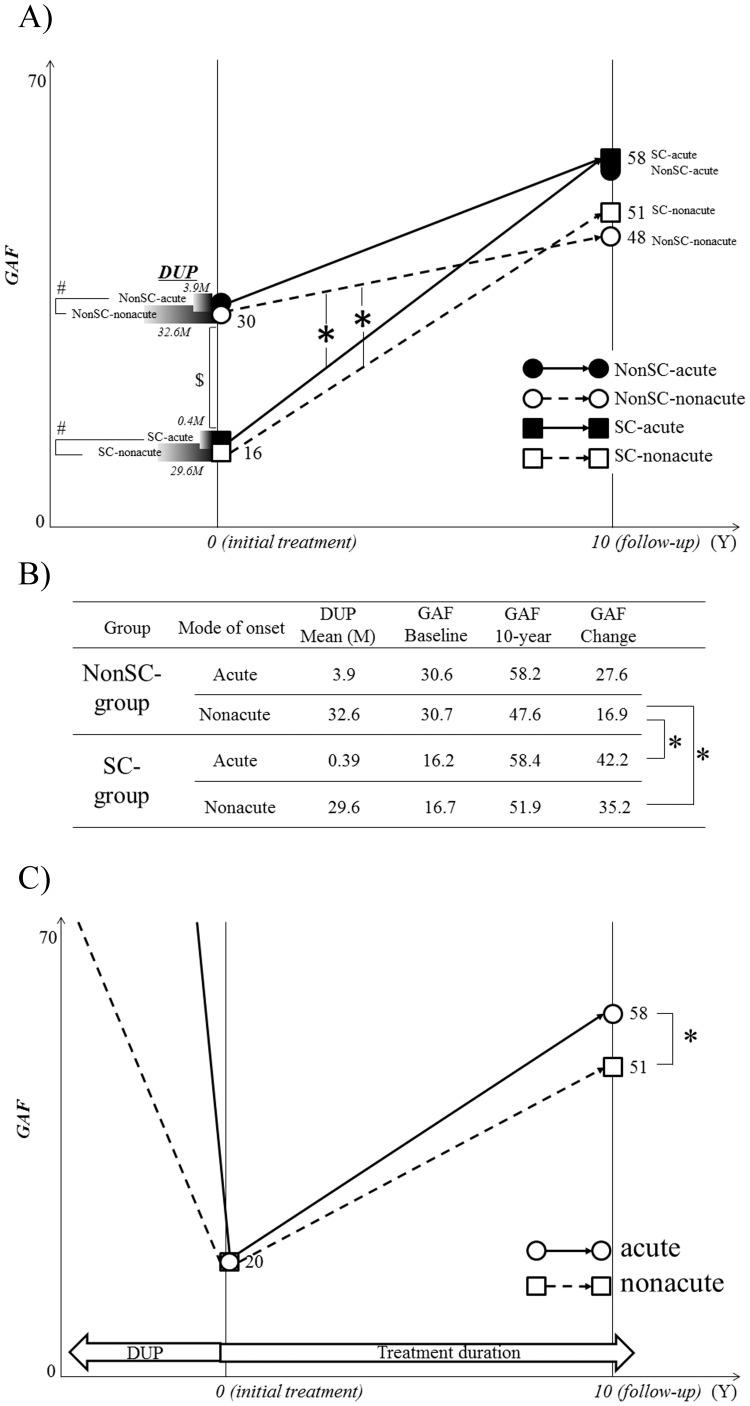
Change in the GAF from treatment intervention to 10-year follow-up point in the 4 subgroups. A)*P<.05 by ANCOVA, indicating lesser increase of GAF in the NonSC-nonacute-group than those in the SC-acute and SC-nonacute groups. # *P*<.05, indicating significant difference in DUP between the two groups. $ *P*<.05 by ANOVA, indicating a lower baseline GAF in the SC group than in the NonSC group. B) This table shows the same data as A), particular for DUP and GAF changes of participants, to support understanding of the manuscript and A). *P<.05 by ANCOVA. C) Comparison in GAF changes between the acute onset and nonacute onset groups. *P<.05 by ANCOVA.

When the change in GAF (follow-up GAF minus baseline GAF) was calculated, the value in the NonSC-nonacute-group was significantly smaller than those in the SC-acute-group and SC-nonacute-group (**Fig. 2A and 2B**).

In these analyses, however, sample sizes, in particular that of the SC-acute-group, were small, possibly causing statistical type II errors. The results in this section, moreover, demonstrated less importance of the degree of initial positive symptoms and indicated that the classification based on MoO could be more valid than the degree of initial positive symptoms in clarifying the effects of the clinical factors during the early stage on the prognosis, in addition to the validity of sample size. Thus changes in GAF in both the acute-group (*N* = 41) and the nonacute-group (*N* = 115) patients, regardless of whether they were the SC and NonSC groups, were additionally analyzed. The results (**Fig. 2C**) revealed that although the nonacute-group had profoundly longer DUP (30.4 months±50.6) than that of the acute-group (1.4 months±5.6), GAFs at baseline were not different between them (20.3 and 20.4, respectively). However, at the 10-year follow-up point, BPRS total (14.8±11.4), negative symptoms (4.1±3.9) and GAF (50.7±16.9) in the nonacute-group were significantly poorer than those in the acute-group (10.6±9.3; 2.4±3.2; 58.3±19.7, respectively: *P*<.05). However, BPRS positive symptoms did not differ between the two groups (acute 3.2±4.1; nonacute 4.5±4.4).

#### 3. Relationship between DUP and prognostic valuables in the SC- and NonSC-combined group

For all follow-up participants, partial correlations between clinical variables and DUP were explored under control for gender, age at onset, MoO and premorbid adjustment. The results revealed that there were significant correlations of DUP with BPRS total (*r* = 0.17, *P*<.01), negative symptoms (*r* = 0.21, *P*<.05) and GAF (*r* = −0.26, *P*<.001), but not positive symptoms (*r* = 0.12, *P*>.05).

## Discussion

To explore the long-term effects of predicting factors prior to treatment intervention on prognosis, the initial positive symptoms severity was stood between predicting factors and prognosis in the present study. Thereby, we examined the effects of exacerbated patterns of psychosis on the following disease courses. Our examination revealed two major findings: (1) clinical processes to first admission in the patients group with relatively long DUP without early intervention could be divided according to several onset patterns, and (2) patients in the NonSC-nonacute-group had the longest DUP among our participants and further presented significantly lesser improvement, indicating that this group could experience refractoriness over long follow-up duration.

### Findings from the Baseline Measurements

The present study demonstrated that longer DUP did not necessarily provide severer positive symptoms in patients with psychosis prior to treatment intervention. This finding was supported by the fact that there was no difference in length of DUP between the SC and NonSC groups, although the SC-group had more profound positive symptoms than did the NonSC-group (**Table 1**). The results suggested that there are other factors which might affect the initial severity of positive symptoms. The finding also might be related to the clinically operational characteristics of DUP; the duration of DUP was defined at its termination by treatment intervention, regardless of changes in the psychotic symptoms during the DUP. Thus we conducted further analysis to examine the effects of MoO and severity of psychosis on the DUP and GAF at the 10-year follow-up (**Table 2**). This analysis revealed that GAF scores did not differ between acute onset and nonacute onset within each SC or NonSC group, but DUP was much longer in the non-acute subgroups in both the SC and NonSC groups. These results indicated that there were several patterns in the psychopathological process from the appearance of the first psychotic symptom to treatment intervention that were dependent on at least MoO and the severity of positive symptoms. It appears that patients in the SC-acute-group who experienced rapid exacerbation of positive symptoms within a very limited period were likely to be taken to the psychiatric hospital immediately after full-brown psychosis, while patients in the NonSC-nonacute-group were likely to be left untreated for at least 2 years, partly because their behavior abnormalities were not readily apparent. These possible disease processes during the period of DUP might explain the lack of relations between DUP and the severity of positive symptoms at treatment intervention in previous studies [12], [20], [21], [22], [23].

### Findings from the Prognostic Measurements

Although none of the measurements at the 10-year follow-up differed significantly among the SC and NonSC acute and nonacute subgroups in this study, all outcomes in the NonSC-nonacute-group tended to be inferior to those of the other three subgroups. In particular, these trends were clearly observed in change of GAF, and indeed the scores were significantly lower in the NonSC-nonacute-group than in the SC/NonSC-acute-groups. The former negative results might reflect the homogenous disease progression into chronicity, in which heterogeneity in symptomatology comes to be reduced gradually over time [22], [24]. The lack of difference, however, in all prognostic values among the four subgroups might be simply due to a low statistical power because of the sample size. Additional analysis in which we examined the simple effect of MoO showed that nonacute mode onset could cause a significant poor total psychopathology and negative symptoms as well as GAF at 10-year follow-up, supporting the greater importance of MoO than initial positive symptoms.

Concurrently, the present study results strongly suggest that patients with acute onset had better subsequent clinical courses than did those with nonacute onset, regardless of the severity of positive symptoms at the early stage (**Fig. 2**). These results were consistent with previous reports showing that acute onset predicted better prognosis, or insidious onset predicted poorer prognosis [12], [13], [25]. Taken together, these results indicate that the initial positive symptoms do not act definitively as a prognosis predictor. This fact may be related to the generally accepted notion that antipsychotics provide greater beneficial effects on positive symptoms in first-episode patients than on other symptoms and/or at other subsequent stages in schizophrenia [26]. Clinical deterioration as a result of repeated relapses following the first episode, on the other hand, may be related to broader psychopathology that includes negative symptoms and cognitive function, in addition to treatment-resistant positive symptoms. In this context, in the field of early intervention for psychosis, the more essential rationale is not simply shortening DUP, leading to attenuation of psychotic symptoms, but attenuating exposures in broader symptom domains.

Our results also showed that patients in the NonSC-nonacute group presented the worst prognosis among the four subgroups, shown by the alteration in the GAF score during the follow-up (**Fig. 2A and 2B**), indicating a greater possible effect of MoO on prognosis relative to initial positive symptoms. Considering the importance of MoO shown in the present study, we conclude that the prolongation of DUP in the NonSC-nonacute group (32.6 months) was due to low impetus of positive symptoms prior to treatment intervention, rather than simple neglect of the relevant psychosis. Thus longer DUP and nonacute onset would play important roles in predicting poor prognosis, and an evaluation of exacerbated pattern during DUP at the first visit could predict the degree of consequent improvement after treatment intervention.

### Generalization of the Present Study

The termination of DUP is generally determined by the treatment intervention for the relevant psychosis, which may be affected by country or psychosocial background and/or medical facilities. This study was conducted in an emergency psychiatric hospital before the establishment of systematic intervention for early psychosis, which may explain the relatively longer DUP of about two years in the present study compared to the DUP in other similar studies. Therefore, the present results included a substantial proportion of patients with long DUP, and might rather reflect a *natural* process that occurs during the early stage of psychosis. Additionally, 5.7% ( = 9/156) of patients committed suicide and 32–51% of patients reached symptoms remission in this cohort, which were quite comparable to the findings reported in other studies (**Table 2**) [27], [28]. When we examined partial correlation between DUP and outcomes in patients as a whole (shown in the last section of the **Results**), we found significant correlations with prognostic measurements other than positive symptoms, which was consistent with previous studies.

On the other hand, the length of DUP in the SC-acute-group was very short, and one might assume that diseases suffered by the patients in this subgroup can meet the criteria of acute and transient psychotic disorders (ATPD), rather than schizophrenia spectrum disorders. Actually, although quite a few patients in this subgroup were suspected of or diagnosed with ATPD at the first admission, most of them were finally diagnosed with schizophrenia through careful examinations. Thus these patients with very short DUP indicated clinically a representative pattern in early psychosis. Taken together, these results suggested that the patients in this study did not constitute a biased sample of patients with some peculiar characteristics, regardless of the fact that our groups included both patients with very short DUP and patients with very long DUP.

### Study Limitations

This study had some limitations. The first is the relatively high drop-out rate. The reason is mainly that substantial numbers of patients were transferred into other hospitals immediately after treatment intervention in our hospital, since CPMC plays a role as an emergency care unit for a broad area. Although baseline data showed quite similar levels of each clinical symptom between the follow-up completers and non-completers, the non-completers who were transferred to other hospitals conceivably needed longer-term admission, implying severer cases. As described in the previous section, we confirmed that our follow-up cases were not substantially different from general schizophrenia patients.

The second limitation is related to study design. The present study did not assign all cases into the SC-group or the NonSC-group, based on the symptoms score at the intervention, but all cases were labeled according to clinical judgment at the patient’s first admission (SC-group or NonSC-group). For example, a patient who went back to his/her home after the initial visit based on his/her family consideration, regardless of admission indication, was possibly included within the NonSC-group.

The third limitation is that clinical scores at the baseline gathered using established clinical measurements (i.e., BPRS and PANSS) were not available. Although the SC-group was surely comprised of patients with more severe illness compared to the NonSC-group, lack of evaluation using the formal scales at baseline might have slightly affected the study outcome.

### Conclusions

The present study demonstrated that neither DUP nor MoO alone could explain the severity of positive symptoms at treatment intervention, but both MoO and DUP could determine several onset patterns of early psychosis. Furthermore, these factors could predict the subsequent natural course of the disease in patients with schizophrenia.
